# Prevalence and Consequences of the Proximal Junctional Kyphosis After Spinal Deformity Surgery: A Meta-Analysis

**DOI:** 10.1097/MD.0000000000003471

**Published:** 2016-05-20

**Authors:** Chunda Yan, Yong Li, Zhange Yu

**Affiliations:** From the 4th Ward of Orthopedics (CY, ZY), the First Affiliated Hospital of Harbin Medical University, Harbin, Heilongjiang; and Department of Orthopaedics (YL), Shanxi Province People's Hospital, Xi’an, China.

## Abstract

The aim of this study was to estimate the prevalence and patient outcomes of proximal junctional kyphosis (PJK) in pediatric patients and adolescents who received surgical interventions for the treatment of a spinal deformity.

Literature was searched in electronic databases, and studies were selected by following précised eligibility criteria. Percent prevalence values of the PJK in individual studies were pooled to achieve a weighted effect size under the random effects model. Subgroup and meta-regression analyses were performed to appraise the factors affecting PJK prevalence.

Twenty-six studies (2024 patients) were included in this meta-analysis. Average age of the patients was 13.8 ± 2.75 years of which 32 ± 20 % were males. Average follow-up was 51.6 ± 38.8 (range 17 ± 13 to 218 ± 60) months. Overall, the percent prevalence of PJK (95% confidence interval) was 11.02 (10.5, 11.5) %; *P* < 0.00001 which was inversely associated with age (meta-regression coefficient: –1.607 [–2.86, –0.36]; 0.014). Revision surgery rate in the patients with PJK was 10%. The prevalence of PJK was positively associated with the proximal junctional angle at last follow-up (coefficient: 2.248; *P* = 0.012) and the change in the proximal junctional angle from surgery to last follow-up (coefficient: 2.139; *P* = 0.014) but not with preoperative proximal junctional angle.

The prevalence of PJK in the children and adolescent patients is 11%. About 10% of those affected require revision surgery.

## INTRODUCTION

Proximal junctional kyphosis (PJK) is a postsurgical spinal deformity that can develop after scoliosis or kyphosis surgery when an instrumented fusion of thoracic vertebrae to lumbar vertebrae causes increased junctional stress over the upper instrumented vertebra.^1^ This complication can develop because of multiple causes including progressive deformity from aging, disruption of the posterior ligament complex, fracture in the uppermost instrumented vertebra, instrumentation failure, degenerative disk disease, and/or facet violation.^2^

The incidence of PJK depends on a variety of factors such as the fusion to sacrum and posterior fusion with segmental instrumentation;^3^ weakening effect caused by muscle dissection and disruption of posterior ligaments; secondary to loss of lumbar lordosis;^4^ incidence of fracture at the upper instrumented vertebra; change in lumber lordosis >30°; pre-existing thoracic kyphosis >30°;^5^ global sagittal balance, and lumbar lordosis.^6^ Moreover, upper instrumented vertebrae of the lower thoracic spine (T10–T12) are reported to bear higher rate of PJK when compared with upper thoracic (T1–T3) segments.^7,8^

Surgery-related factors posing risk of PJK include combined anterior-posterior surgery, thoracoplasty, upper instrumented vertebra at T1–T3, riser, the disruption of the spinal posterior tension band because of intraoperative lesions of the inter- and supra-spinous ligament, and due to an excessive dissection of the para-spinal muscles during spinal exposure especially in the region immediately cephalad to the instrument implant.^1,6^ Owing to such factors, the PJK prevalence reported in different studies is considerably higher with about 66% of PJK develops within 3 months and 80% within 18 months after surgery.^2^

In more serious cases, PJK can cause neurological impairment that may necessitate reoperation involving decompression of the spinal cord.^9^ Several surgical interventions are proposed to reduce PJK occurrence including preservation of the superjacent facets and supraspinous ligament, performing laminectomy to intervene the upper most instrumented vertebrae (UIV) at a level that must not lack posterior column deficiency, listhesis, rotation, or junctional kyphosis; and avoiding a UIV location at the apex of the deformity in either coronal or sagittal plane.^10–12^

In literature, data pertaining to the prevalence of PJK is provided mainly by retrospective studies. In these studies, the PJK prevalence following spinal deformity surgeries range from 0% to 55%. Because of the multifactorial etiology of PJK, it is important to study the PJK prevalence in various subgroups of patients and to explore the avenues of risk assessment. The present study was designed to systematically review the relevant studies which reported the prevalence of PJK after surgical interventions in children and adolescents with spinal deformity and have a pooled analysis of the prevalence in the overall population and subgroups of the patients. An attempt is also made to identify risk factors for the development of PJK by applying meta-regression analyses to the available data.

## MATERIAL AND METHODS

This study was performed by following Preferred Reporting Items for Systematic Reviews and Meta-Analyses (PRISMA) guidelines.^13^ The important steps of method used in this study are presented in Table 1. As this study is a meta-analysis research with published data as materials, it does not need approval from the institutional review board.

**TABLE 1:**
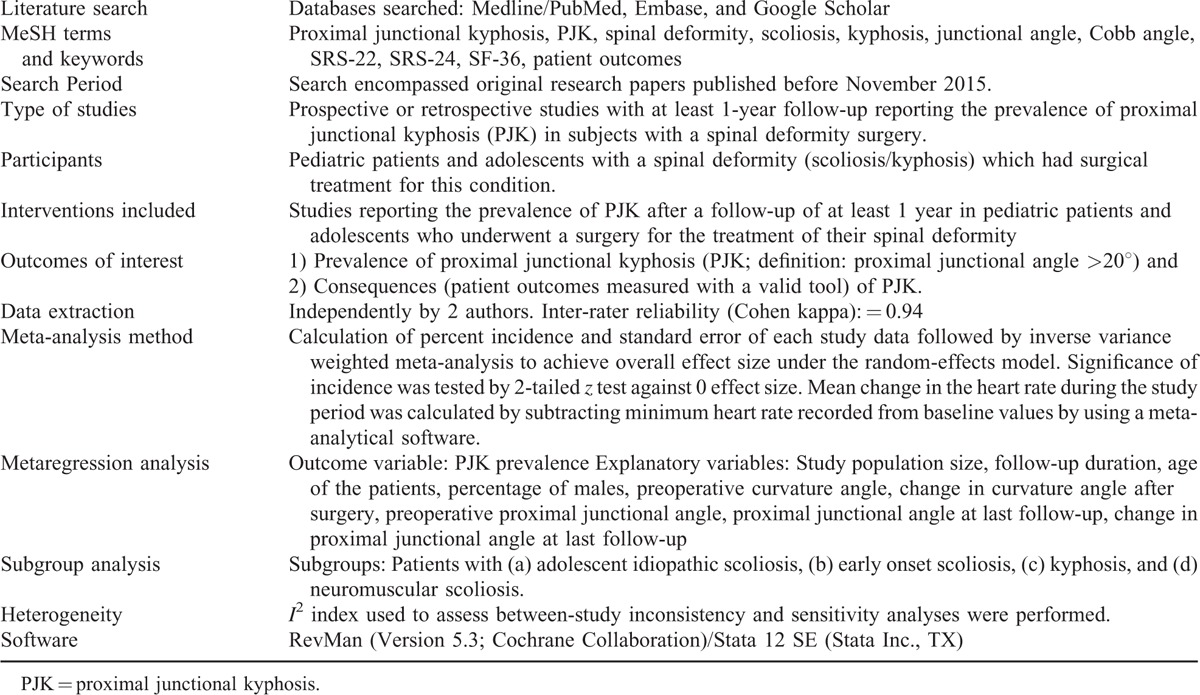
Important Features of the Method Used for the Present Study

## RESULTS

**FIGURE 1:**
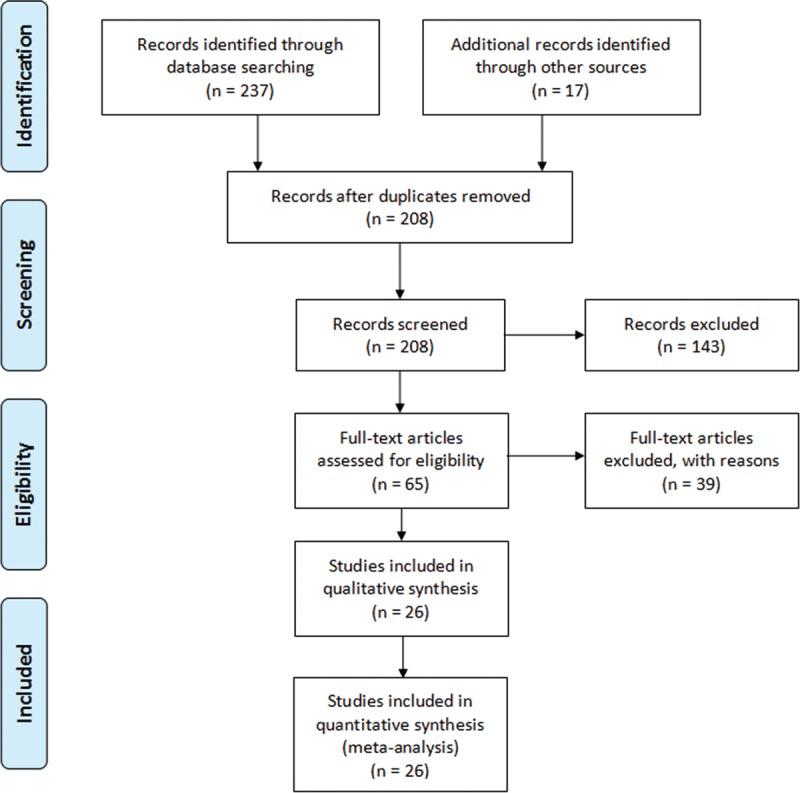
A flowchart of study screening and selection process.

Twenty-six studies^14–38^ fulfilled the eligibility criteria and were included in this meta-analysis (Figure 1). Of these, 3 were prospective^26,34,39^ and the rest were retrospective in design. From the included studies, data of 2024 patients is used in this meta-analysis. Average follow-up was 51.6 ± 38.8 (range 17 ± 13–218 ± 60) months. Average age of the patients was 13.8 ± 2.75 (rang 4.8 ± 2.1–18.6 ± 3) years and 32 ± 20 % were male in this sample population.

Twelve studies recruited adolescent idiopathic scoliosis patients, 5 studies recruited early onset scoliosis patients, 4 studies recruited kyphosis patients, 2 studies recruited neuromuscular scoliosis patients, and 1 study each recruited congenital kyphosis, congenital scoliosis, and scoliosis secondary to skeletal dysplasia patients.

**FIGURE 2:**
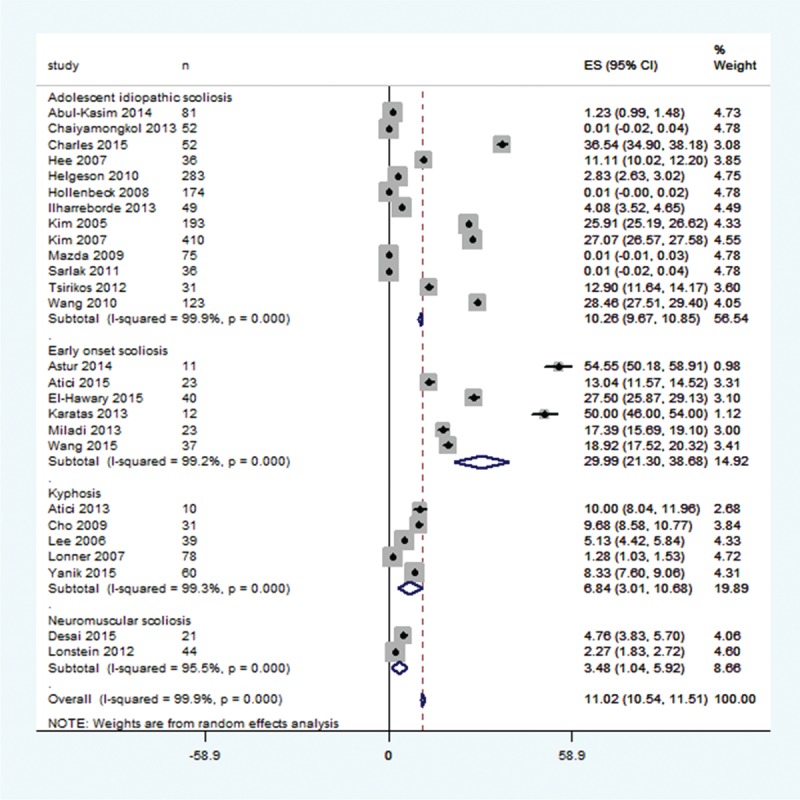
A forest graph showing the results of a pooled analysis of the percent prevalence of proximal junctional kyphosis in the included studies and overall effect size according to conditions.

Overall, the prevalence of PJK at the latest follow-up was statistically significantly higher against the zero effect size. Random effects meta-analysis revealed that the percent prevalence of PJK (95% confidence interval; CI) was 11.02 (10.5, 11.5); %; *P* < 0.00001 (Figure 2). The percent prevalence (95% CI) of PJK in subgroups was: early onset scoliosis (29.99 [21.3, 36.66] %; *P* < 0.00001), Adolescent idiopathic scoliosis (10.3 [9.7, 10.8] %; *P* < 0.00001), kyphosis (6.84 [3.01, 10.68] %; *P* < 0.00001), and neuromuscular scoliosis (3.48 [1.04, 5.92] %; *P* = 0.005). In the patients with PJK, the revision surgery rate was 10% (11 studies data).

In the meta-regression analyses (Table 2), the prevalence of PJK was inversely associated with age (coefficient: –1.61; *P* = 0.014) but was not significantly associated with the number of patients in a study, the percentage of males in a study, and the follow-up duration. The prevalence of PJK was positively associated with the proximal junctional angle at last follow-up (coefficient: 2.248; *P* = 0.012) and the change in the proximal junctional angle from surgery to last follow-up (coefficient: 2.139; *P* = 0.014) but was not significantly associated with preoperative proximal junctional angle, the preoperative curvature angle, or the change of curvature angle at latest follow-up.

**TABLE 2:**
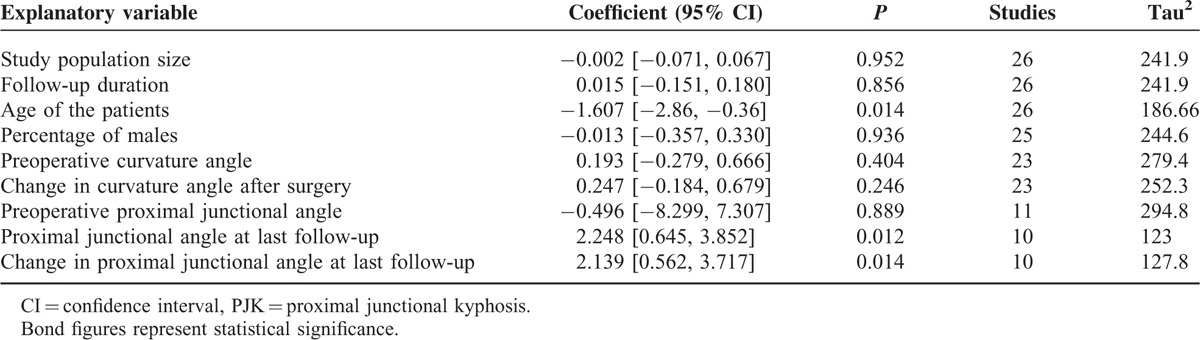
Metaregression Outcomes Explaining the Factors Affecting the PJK Prevalence

## DISCUSSION

We have found that in children and adolescents who underwent a scoliosis or kyphosis surgery, the prevalence of PJK is 11% (range 0–55%) in this sample of 2024 patients at the latest follow-up which was 52 months on average. Subgroup, early onset scoliosis, exhibited highest prevalence of PJK (30%). The prevalence was inversely associated with age but positively associated with change in the proximal junctional angle at latest follow-up but not with the preoperative proximal junctional angle. About 10% of those who developed PJK required revision surgery.

In adults, the PJK prevalence range between 5% and 46% of the subjects of spinal instrumentation for skeletal deformity with majority of the studies have observed PJK prevalence between 20% and 40%.^2^ In the present synthesis, only 5 of 26 included studies observed the PJK prevalence over 20% and rest of these studies reported the prevalence under 20%. This may indicate that in children and adolescents, the PJK prevalence is less than in adults, but this requires confirmation in a systematic review of adult PJK prevalence.

In adults, the prevalence of PJK (≥20°) in primary idiopathic/degenerative scoliosis patients did not require revision surgery in many studies (e.g., Bridwell et al)^39^ and if so, patients with PJK requiring revision are often older, have higher postoperative lumbar lordosis, and larger sagittal balance corrections than patients without PJK.^40^ In the present study, 11 of the included studies reported whether revision surgery in patients with PJK was carried out or was not required. In these 11 studies, the revision rate was 10%. However, if it is assumed that in remaining studies no revision surgery was required, then the rate of revision surgery should had reduced.

Among the included studies, many authors attempted to identify the risk factors associated with PJK development. Among these, El-Hawary et al^22^ found that in comparison with subjects without PJK, subjects with PJK had significantly greater cervical lordosis and proximal junctional angle at final follow-up. Helgeson et al^24^ noted a trend toward a decrease in PJK with placement of hooks at the upper instrumented vertebra than with screws. Moreover, they suspected that increased BMI may be a risk factor for PJK. Kim et al^28,29^ identified thoracoplasty, larger preoperative thoracic hyperkyposis angle, greater immediate postoperative thoracic kyphosis, hybrid instrumentation, decrease in the Cobb angle, and male gender as risk factors for PJK development. Wang et al^4^ identified thoracoplasty, distraction of correction, using screws for upper vertebra fixation, corrected angle of thoracic vertebrae, and fused lumbar vertebrae below L2 as the risk factors for PJK development. Wang et al^37^ reported that PJK was associated with greater postoperative segmental kyphosis, grater proximal junctional angle, screw malposition on the UIV, and the location of hemivertebra on the lower thoracic/thoracolumbar region.

To evaluate the patient outcomes at final follow-up, SRS-24 questionnaire was used only by a couple of the included studies. In 1 study,^28^ in which 78% patients without PJK and 70% patients with PJK completed questionnaires, there was no significant difference; the PJK group had a total score of 98 and self-image subscales of 23 whereas control patients had total score of 93 and self-image subscale of 22. In another study^29^ also, SRS-24 scores at 2 years follow-up were not significantly different between PJK affected and normal subjects. However, Kim et al^29^ have cautioned that SRS-24 may not be a sensitive instrument in examining the patients outcomes in young patients as usually young patients seldom complain of spinal pain unless more severe conditions such as the degeneration of the cervicothoracic junction develop.

Hollenbeck et al^25^ found that total health-related quality-of-life outcomes at an average of 8 years’ follow-up were similar for patients with normal and increased proximal junctional flexion. Desai et al^21^ used SRS-22 questionnaire and observed a last follow-up score of 4.3 ± 0.5 overall and these patients had a SF-36 PCS score of 46.6 ± 10.6. In their study, only 1 patient developed PJK who had a SF-36 PCS score of 34.2. None of the other studies attempted to measure patient outcomes at last follow-up, and therefore, this important aspect should be considered in the future studies.

Thus, the current evidence do not indicate any major clinical consequence of PJK in terms of reoperations and patients outcomes (SRS-24/SF-36 scores etc.). It is also observed that chances of PJK development decrease much after 2 years of surgery and even if PJK exists radiographically at 7.3 years after surgery, its impact on the patient's perception of outcome could be minimal.^28^ However, because of the scarcity of long-term appraising patient outcomes for PJK in children and adolescents, future studies should include patient outcome measures in the designs of the trials.

## CONCLUSIONS

Overall incidence of the proximal junctional kyphosis in the pediatric and adolescent patients was estimated at 11% with a range of 0–55%. The revision surgery rate was 10% in the patients who developed PJK. The prevalence was inversely associated with age but positively associated with change in the proximal junctional angle at latest follow-up but not with the preoperative proximal junctional angle. Future studies should address patient outcome measures in their designs.
